# Hispolon suppresses metastasis via autophagic degradation of cathepsin S in cervical cancer cells

**DOI:** 10.1038/cddis.2017.459

**Published:** 2017-10-05

**Authors:** Min-Chieh Hsin, Yi-Hsien Hsieh, Po-Hui Wang, Jiunn-Liang Ko, I-Lun Hsin, Shun-Fa Yang

**Affiliations:** 1 Institute of Medicine, Chung Shan Medical University, Taichung, Taiwan; 2 Institute of Biochemistry, Microbiology and Immunology, Chung Shan Medical University, Taichung, Taiwan; 3 Department of Obstetrics and Gynecology, Chung Shan Medical University Hospital, Taichung, Taiwan; 4 Inflammation Research & Drug Development Center, Changhua Christian Hospital, Changhua, Taiwan; 5 Department of Medical Research, Chung Shan Medical University Hospital, Taichung, Taiwan

## Abstract

Hispolon, a phenolic compound isolated from *Phellinus igniarius*, induces apoptosis and anti-tumor effects in cancers. However, the molecular mechanism involved in hispolon-mediated tumor-suppressing activities observed in cervical cancer is poorly characterized. Here, we demonstrated that treatment with hispolon inhibited cell metastasis in two cervical cancer cell lines. In addition, the downregulation of the lysosomal protease Cathepsin S (CTSS) was critical for hispolon-mediated suppression of tumor cell metastasis in both *in vitro* and *in vivo* models. Moreover, hispolon induced autophagy, which increased LC3 conversion and acidic vesicular organelle formation. Mechanistically, hispolon inhibited the cell motility of cervical cells through the extracellular signal-regulated kinase (ERK) pathway, and blocking of the ERK pathway reversed autophagy-mediated cell motility and CTSS inhibition. Our results indicate that autophagy is essential for decreasing CTSS activity to inhibit tumor metastasis by hispolon treatment in cervical cancer; this finding provides a new perspective on molecular regulation.

Cervical cancer remains the second most common cancer affecting women worldwide, and although early screening might reduce mortality rates,^1^ more than 260 000 women die of cervical cancer annually.^2^ Human papillomaviruses are extremely common worldwide, and the subtypes 16 and 18 are major causes (70%) of cervical cancer. The risk factors for cervical cancer include smoking, multiple sexual partners, and viral and other infections.^3^

The hallmarks of cancer include the activation of invasion and metastasis, which is the primary cause of mortality in most cancers.^4^ Therefore, the suppression of tumor metastasis is a critical therapeutic target of cancer. Cathepsins play a critical role in cancer metastasis. They are highly expressed in human cancer cells, particularly in invasive tumor cells. Each member of the Cathepsin family performs different functions in tumor metastasis. Imbalance between the cathepsins and cysteine proteinase inhibitors causes metastasis of cancer cells.^5^ Moreover, patients harboring tumors with positive Cathepsin expression exhibit poor outcomes. Therefore, this superfamily has been suggested as a prognosis marker.^6^ Among the family members, Cathepsin S (CTSS) is capable of degrading the extracellular matrix and promoting cell metastasis.^7^ CTSS can regulate breast-to-brain metastasis. In addition, breast cancer patients with high CTSS expression display a poor prognosis.^8^

Autophagy is a critical catabolic process for damaged organelles, unfolded proteins, and bulk cytosol in double-membrane vesicles; autophagosomes capture intracellular cargo and fuse with lysosomes, followed by degradation.^9^ Degradation generates energy, and the degraded cargo is shuttled to the cytoplasm for recycling, which promotes cell survival.^10, 11^ Several factors may induce autophagy, including starvation, low oxygen saturation, hormonal stimulation, and damaged organelle accumulation.^12^ High levels of autophagy can induce autophagic cell death.^13^ However, some studies have suggested that suitable levels of autophagy promote cell survival.

Hispolon (6-(3,4-dihydroxy-phenyl)-4-hydroxy-hexa-3,5-dien-2-one; C_12_H_12_O_4_) is a phenolic compound isolated from *Phellinus igniarius*^14^(Figure 1a), and it has anti-inflammatory, anti-proliferative, antioxidant, and anti-tumor effects.^15^ Hispolon may induce G0/G1 cell cycle arrest and apoptosis in human leukemia, nasopharyngeal carcinoma, and hepatocellular carcinoma cells^15, 16, 17^ and enhance the expression of the cancer-suppressor gene p53 in lung cancer cells.^14^ Furthermore, hispolon can inhibit tumor metastasis in human hepatoma through suppression of MMP-2/9 and urokinase-plasminogen expression.^18^ Moreover, hispolon may induce autophagy in hepatocellular carcinoma and breast cancer cells.^19^ However, the relationship between hispolon and cervical cancer is poorly understood. Therefore, this study examined the effects of hispolon on human cervical cancer cells. Our findings indicate that hispolon is a pharmaceutical compound that exerts anti-metastatic effects in cervical cancer through an autophagy-dependent mechanism.

## Results

### Hispolon inhibits cell migration and invasion in cervical cancer cells

**Figure 1:**
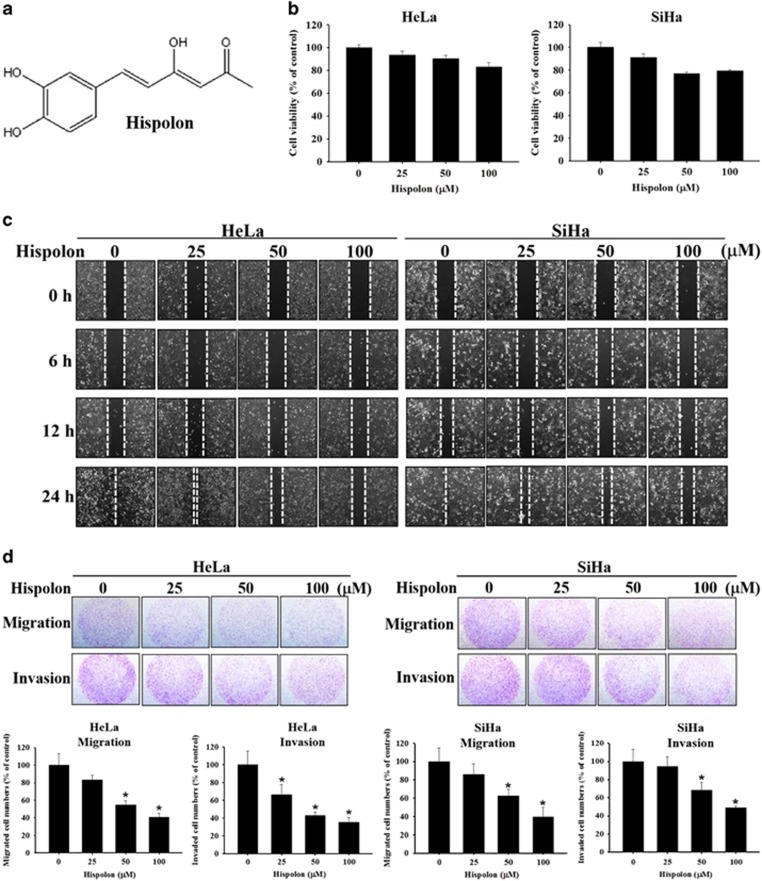
Effects of hispolon on cell viability, migration and invasion in cervical cancer cell lines. (**a**) The chemical structure of Hispolon. (**b**) HeLa and SiHa cells were seeded onto 24-well plates and treated with hispolon (0, 25, 50, 100 *μ*M) for 24 h and then analyzed by MTT assay. (**c**) HeLa and SiHa cells were treated onto six-well plates and draw a line between cells and cells then observed the ability of healing in 6, 12, 24 h on various hispolon concentrations by microscope. (**d**) HeLa and SiHa cells were seeded onto 24-well plates and treated with hispolon (0, 25, 50, 100 *μ*M) for 24 h and then analyzed by Boyden chamber assay. (The values represented the mean±S.D. from three determinations per condition repeated three times. *P<0.05 compared with untreated.)

To investigate the effects of hispolon on cervical cancer cell viability, HeLa cells and SiHa cells were treated with hispolon (0, 25, 50, 100 *μ*M) for 24 h and were analyzed through an MTT assay. The data revealed that high-dose hispolon only reduced cell viability by 20% in cervical cancer cells (Figure 1b). To assess the anti-metastatic effects of hispolon in cervical cancer, a wound healing assay and a migration/invasion assay were performed using a culture insert and Boyden chamber, respectively. The results revealed that hispolon inhibited the migration and invasion abilities of cervical cancer cells in a dose-dependent manner (Figures 1c and d).

### Hispolon inhibits metastasis by reducing CTSS expression in cervical cancer cells

**Figure 2:**
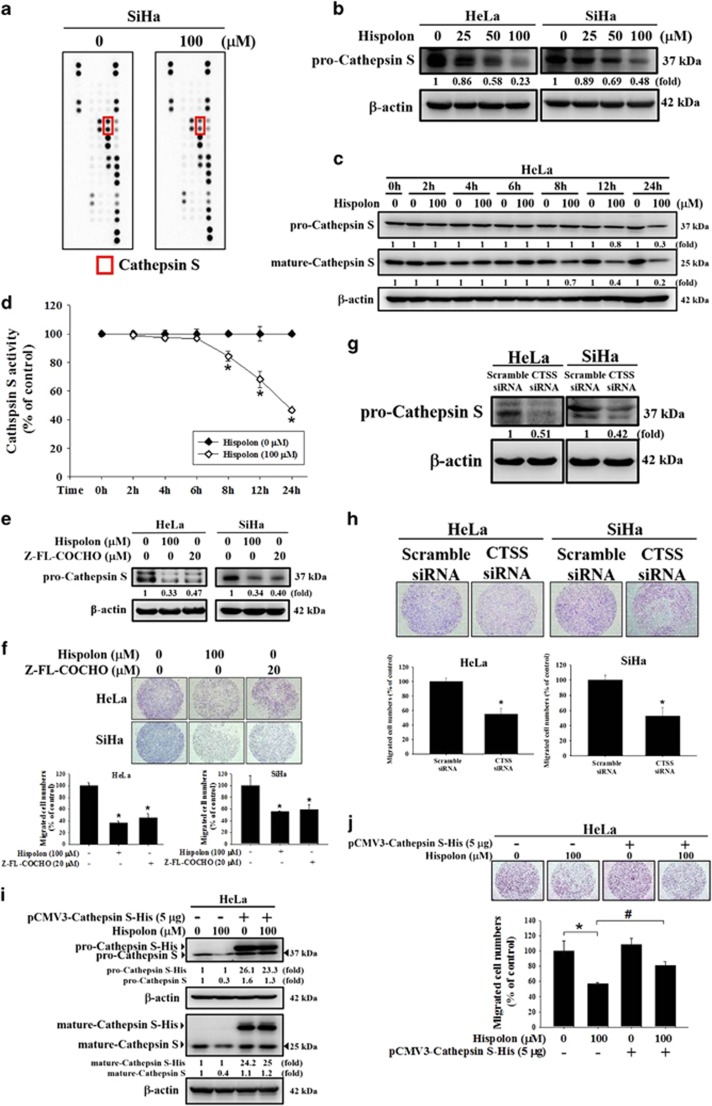
Effects of hispolon on proteinases expression which influenced the ability of metastasis in cervical cancer cells. (**a**) Collected the cell protein lysate and detected the protein expression by Human Proteinases Array. (**b**) The cells were seeded on 6 cm plates and treated with hispolon (0, 25, 50, 100 *μ*M) for 24 h. Then the cell protein lysate was collected and analyzed by western blot assay. (**c**,**d**) After treatment with hispolon (0,100 *μ*M) in 0, 2, 4, 6, 8, 12 and 24 h. The cell lysates were collected in cell lysis buffer and analyzed by Cathepsin S activity assay. (**e**,**f**) The cells were treated with hispolon (100 *μ*M) or Cathepsin S inhibitor Z-FL-COCHO (20 *μ*M) for 24 h, then analyzed by western blot and migration assay. (**g**,**h**) HeLa and SiHa cells transfected with the siRNA of Cathepsin S for 48 h and analyzed by western blot and migration assay. (**i**,**j**) HeLa cells transfected with pCMV3-CTSS-His plasmid 5 *μ*g for 6 h and treated with hispolon for 24 h, then analyzed by Western blot and migration assay. (The values represented the mean±S.D. from three determinations per condition repeated three times. *P<0.05 compared with untreated. ^#^*P*<0.05 compared with hispolon (100 *μ*M))

To identify the protease participating in hispolon-mediated inhibition of metastasis, a human protease array was performed in SiHa cells. The data showed that hispolon markedly reduced CTSS expression (Figure 2a). Through a western blot assay, we demonstrated that hispolon (0, 25, 50, 100 *μ*M) reduced CTSS expression in a dose-dependent manner (Figure 2b). The mature form of CTSS also inhibited by hispolon (Supplementary Figures S1 and S2). HeLa cells were treated with Hispolon (100 *μ*M) in a time-course-dependent manner (0, 2, 4, 6, 8, 12, 24 h). Comparing with untreated cells, Hispolon (100 *μ*M) accelerating Cathepsin S degradation in 8, 12 and 24 h. In Figure 2d, we also detected the activity of Cathepsin S in various time-course manner by activity assay. The data showed that Cathepsin S activity significantly decreased in 8, 12 and 24 h when compared with untreated cells (Figures 2c and d). The CTSS inhibitor Z-FL-COCHO was used to investigate the role of CTSS in cervical cancer metastasis. Z-FL-COCHO inhibited CTSS expression in both cervical cancer cell lines and reduced the migration of cervical cancer cells (Figures 2e and f). CTSS-targeting siRNA also inhibited the migration ability of HeLa and SiHa cells (Figures 2g and h). Furthermore, silencing CTSS then treated with hispolon that showed enhancing the effect of mature-CTSS inhibition (Supplementary Figure S1). Besides, overexpression of CTSS presented that reversed hispolon-inhibited pro-CTSS and mature-CTSS protein level and migration in HeLa cells (Figures 2i and j). These results prove that CTSS plays a critical role in such effect of hispolon on cervical cancer metastasis.

### Hispolon induces autophagy in cervical cancer cells

**Figure 3:**
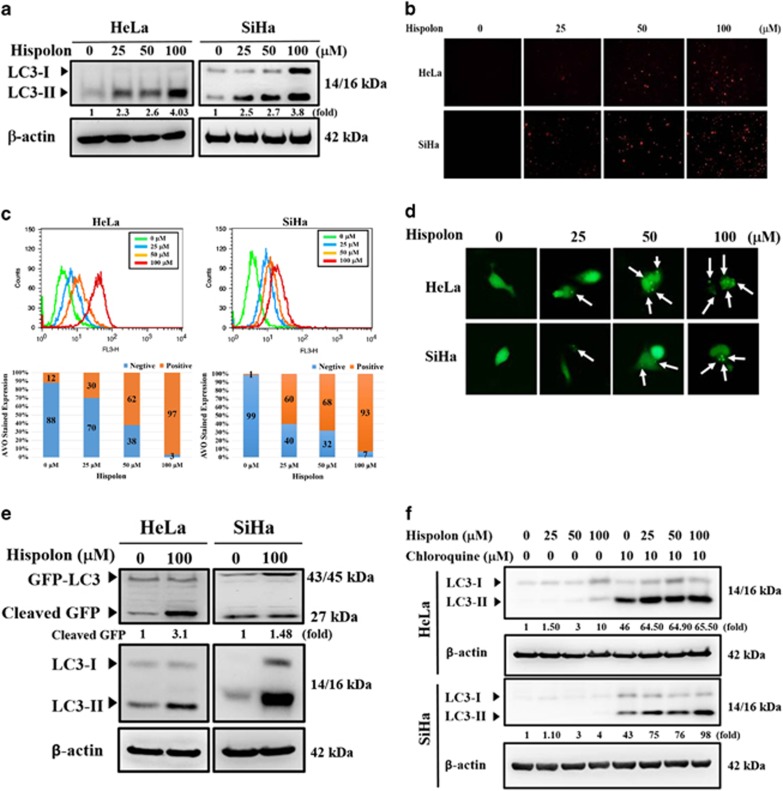
Effects of hispolon-induced autophagy in cervical cancer cells. (**a**) Transformation of LC3-I to LC3-II after HeLa and SiHa cells were treated by various hispolon concentrations (0, 25, 50 and 100) for 24 h and the results analyzed by western blot. (**b**,**c**) To detect the AVOs by acridine orange stained after hispolon treatment and analyzed by microscopy or flow cytometry. (**d**) GFP-LC3 dots were observed after the cells transfected with the plasmid and hispolon treatment. (**e**,**f**) To determine that normal flux autophagy was induced by hispolon in cervical cancer cells. These results were analyzed by western blot

To investigate autophagy induced by hispolon, a dose-dependent experiment was performed through a western blot assay, acidic vesicular organelle (AVO) development, and autophagosome analysis. Hispolon increased LC3-II expression and AVO development in a dose-dependent manner (Figures 3a–c). Furthermore, Hispolon induced green fluorescent protein (GFP)-LC3 puncta, suggesting that hispolon elicits autophagosome formation in cervical cancer cells (Figure 3d). To assess the autophagic flux or block in autophagy induced by hispolon, GFP-LC3 cleavage assay and lysosome inhibitions were performed. As shown in Figure 3e, hispolon increased cleaved GFP expression in cervical cancer cells. Moreover, the lysosomal inhibitor chloroquine blocked the hispolon-mediated LC3-II turnover in HeLa and SiHa cells (Figure 3f). These results demonstrate that hispolon induced autophagic flux in cervical cancer cells.

### Hispolon inhibits the migration of cervical cancer cells by activating autophagy

**Figure 4:**
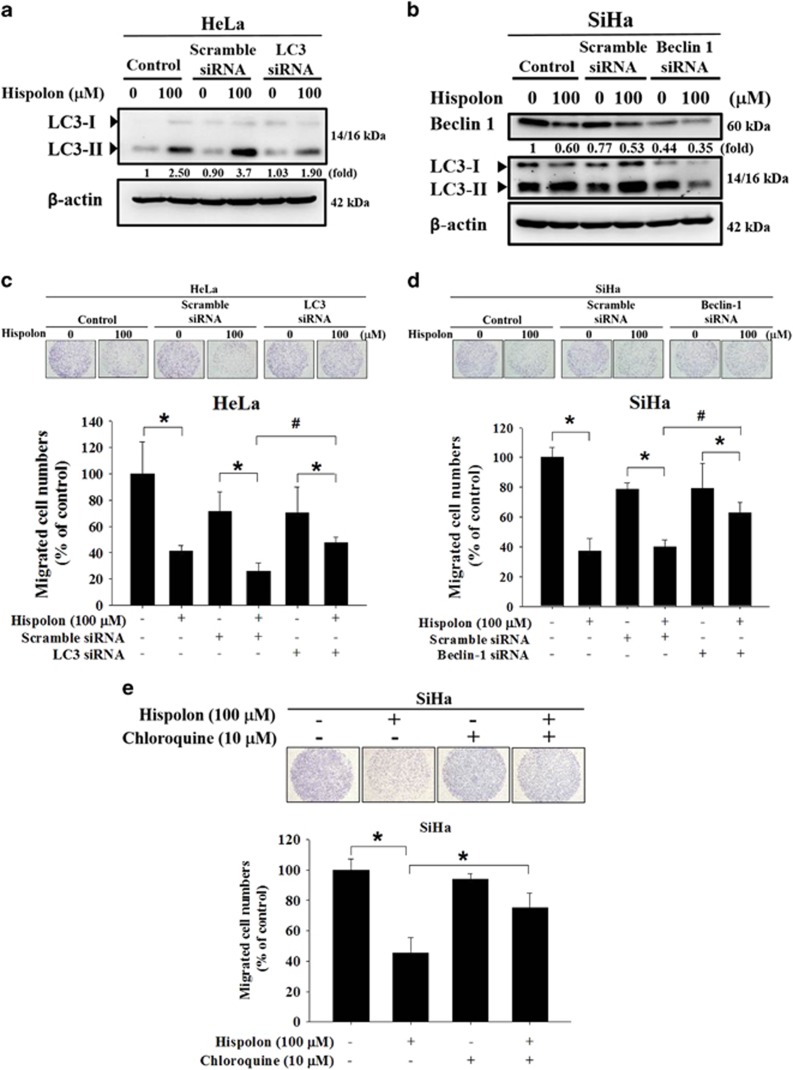
Effects of hispolon-induced autophagy on metastasis in cervical cancer cells. (**a**–**d**) HeLa and SiHa cells were transfected with LC3 or Beclin 1 siRNA for 48 h and then treated with hispolon for 24 h. The results were determined by western blot and migration assay. (**e**) SiHa cells were treated with hispolon and co-treated with CQ and analyzed by migration assay. (The values represented the mean±S.D. from three determinations per condition repeated three times. *P<0.05 compared with untreated. ^#^compared with scramble siRNA which treated with hispolon)

To assess the role of autophagy in hispolon-inhibited metastasis, LC3-B and beclin-1-targeted siRNA were used to inhibit autophagy in HeLa cells and SiHa cells, respectively (Figures 4a and b). Compared with scramble siRNA, LC3 siRNA and beclin-1 siRNA significantly mitigated hispolon-mediated migration inhibition in HeLa and SiHa cells, respectively (Figures 4c and d). Furthermore, chloroquine reversed hispolon-inhibited migration in SiHa cells (Figure 4e). These results suggest that autophagy participates in hispolon-induced migration inhibition.

### Hispolon inhibits CTSS expression through an autophagy-lysosomal system in cervical cancer cells

**Figure 5:**
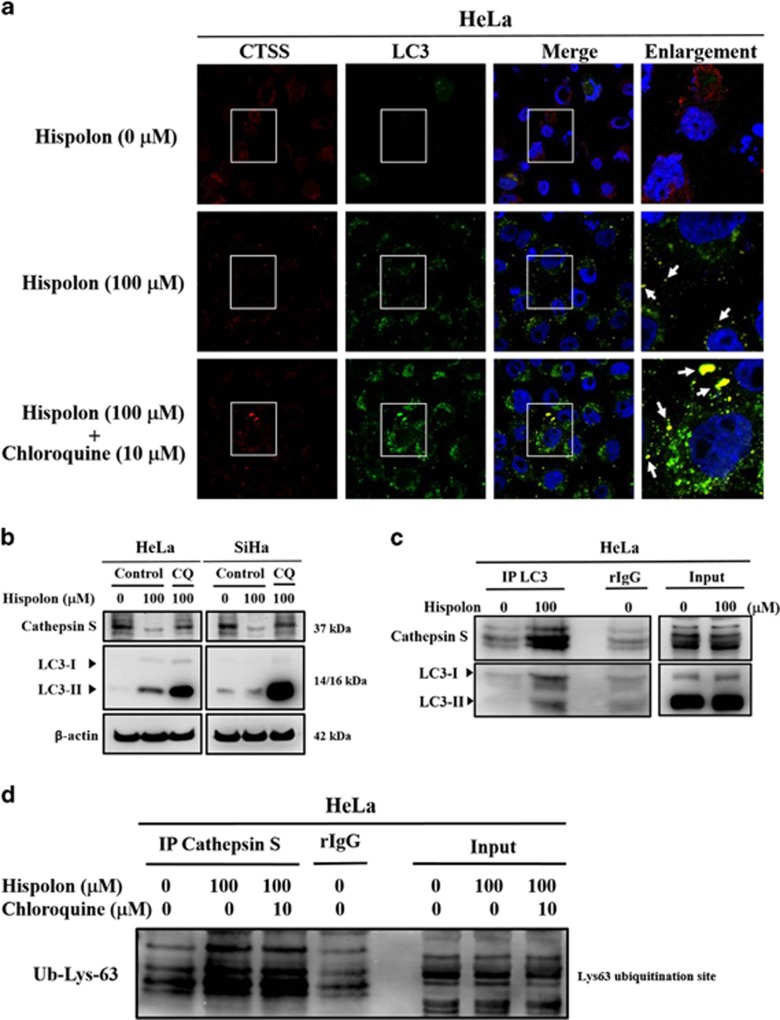
The interaction between Cathepsin S, LC3 and Ubiquitin. (**a**) HeLa cells were pre-treated with CQ for 1 h and co-treated with hispolon for 23 h. Co-stained with LC3 and CTSS antibody and observed by confocal microscopy. (**b**) HeLa and SiHa cells were pre-treated with CQ for 1 h and co-treated with hispolon for 23 h then analyzed by western blot. (**c**) After treating the cells with hispolon, detecting that LC3 interacted with CTSS by Co-IP. (**d**) The cells were treated with hispolon and co-treated with CQ then detecting the interaction between Cathepsin S and Ubiquitin by CO-IP

To further clarify the relationship between CTSS and autophagy, confocal microscopy was used to investigate the localizations of CTSS and the autophagosome in hispolon-treated cervical cancer cells. As shown in Figure 5a, CTSS and the autophagosome were colocalized in HeLa cells treated with hispolon. Furthermore, chloroquine increased the colocalization of CTSS and the autophagosome. The western blot assay also demonstrated that chloroquine abolished the hispolon-mediated decrease in pro-CTSS and mature-CTSS expression (Figure 5b and Supplementary Figure S2). To investigate whether CTSS is captured by the autophagosome, a co-immunoprecipitation assay was performed using LC3 antibodies. As shown in Figure 5c, CTSS was co-immunoprecipitated with LC3 from hispolon-treated HeLa cells. Autophagy involves the ubiquitinated protein degradation pathway.^20^ Hispolon induced CTSS interaction with ubiquitination in HeLa cells (Figure 5d). These data demonstrate that hispolon inhibited CTSS through an autophagic degradation pathway.

### ERK activation by hispolon induces autophagy and the inhibition of cell migration and invasion in cervical cancer cells

**Figure 6:**
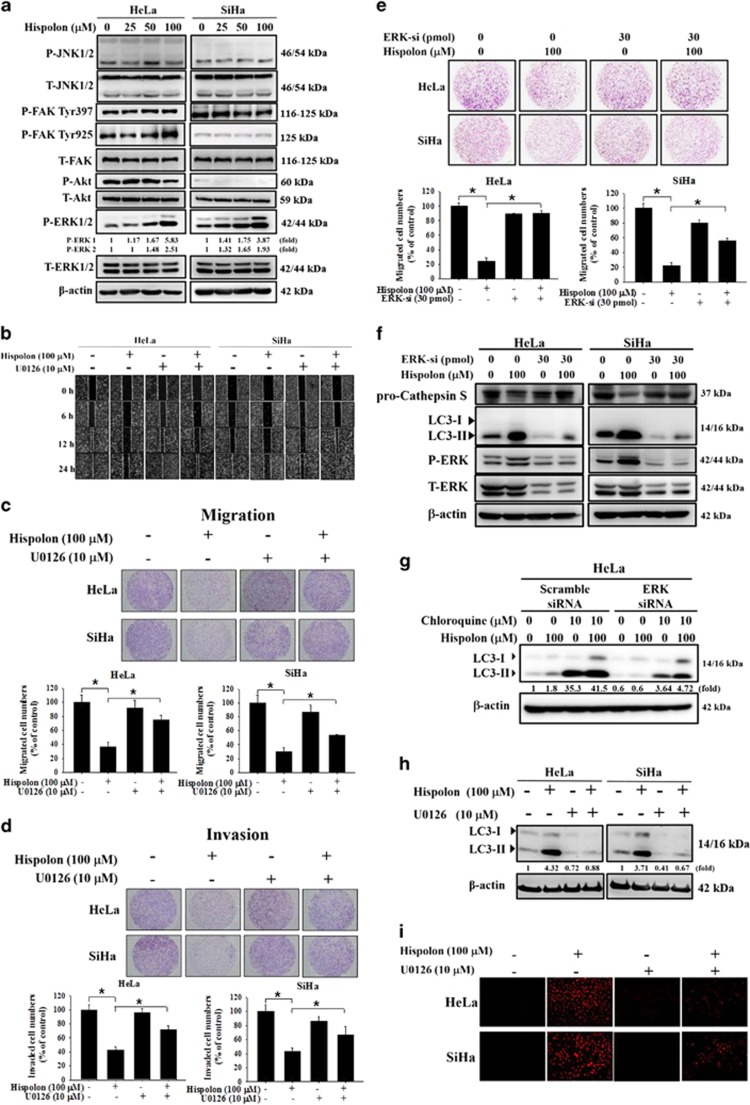
Hispolon-mediated autophagy and suppressed cell metastasis via P-ERK pathway in cervical cancer cells. (**a**) HeLa and SiHa cells were treated with various hispolon concentrations and analyzed MAPK pathway by western blot. (**b**) The cells were pre-treated with U0126 and co-treated with hispolon then the results were observed by wound healing assay. (**c**,**d**) HeLa and SiHa cells were treated as the same as (**b**) and the results were determined by migration and invasion assay. (**e**,**f**) The cells were transfected with ERK-siRNA for 48 h and treated with hispolon for 24 h. The results were detected by migration assay and western blot. (**g**) HeLa cells were transfected with ERK-siRNA for 48 h and pre-treated with chloroquine 1 h and co-treated with hispolon for 23 h then analyzed by western blot assay. (**h**) The cells were pre-treated with U0126 for 1 h and then co-treated with hispolon for 23 h. These results were detected by western blot. (**i**) HeLa and SiHa cells were treated with the MEK inhibitor U0126 for 1 h and co-treated with hispolon. To detect the AVO and observed by fluorescent microscopy. (The values represented the mean±S.D. from three determinations per condition repeated three times. *P<0.05)

The mitogen-activated protein kinase (MAPK) signaling pathway plays a crucial role in cancer cell metastasis.^21, 22^ The data showed that hispolon decreased P-AKT and increased P-FAK Tyr925 levels in HeLa cells but did not alter the activities of JNK, FAK, and Akt signaling pathways in SiHa cell line, whereas hispolon activated the extracellular signal-regulated kinase (ERK) signaling pathway in both HeLa and SiHa cell lines (Figure 6a). The MEK/ERK inhibitor U0126 was used to clarify the effect of ERK activation on the migration and invasion of cervical cancer cells after treatment with hispolon. Wound healing and the Boyden chamber assay revealed that after pretreatment with U0126, the inhibitory effect of hispolon on the migration and invasion of HeLa and SiHa cells was significantly reversed (Figures 6b–d). Furthermore, the silencing of ERK mitigated hispolon-inhibited CTSS protein level and cell migration (Figures 6e and f). It has been reported that ERK activation can trigger autophagy.^23, 24^ Therefore, we examined the role of ERK in hispolon-induced autophagy. As shown in Figure 6g, silence of ERK mitigated the LC3-II accumulation induced by hispolon and chloroquine, suggesting that ERK plays an important role in triggering autophagy signaling. U0126 inhibited hispolon-elicited AVO development and LC3-II upregulation (Figures 6h and i). These results demonstrate that hispolon induced the inhibition of metastasis through the ERK/autophagy pathway in cervical cancer cells.

### Hispolon suppresses cell metastasis in a SiHa xenograft tumor model

**Figure 7:**
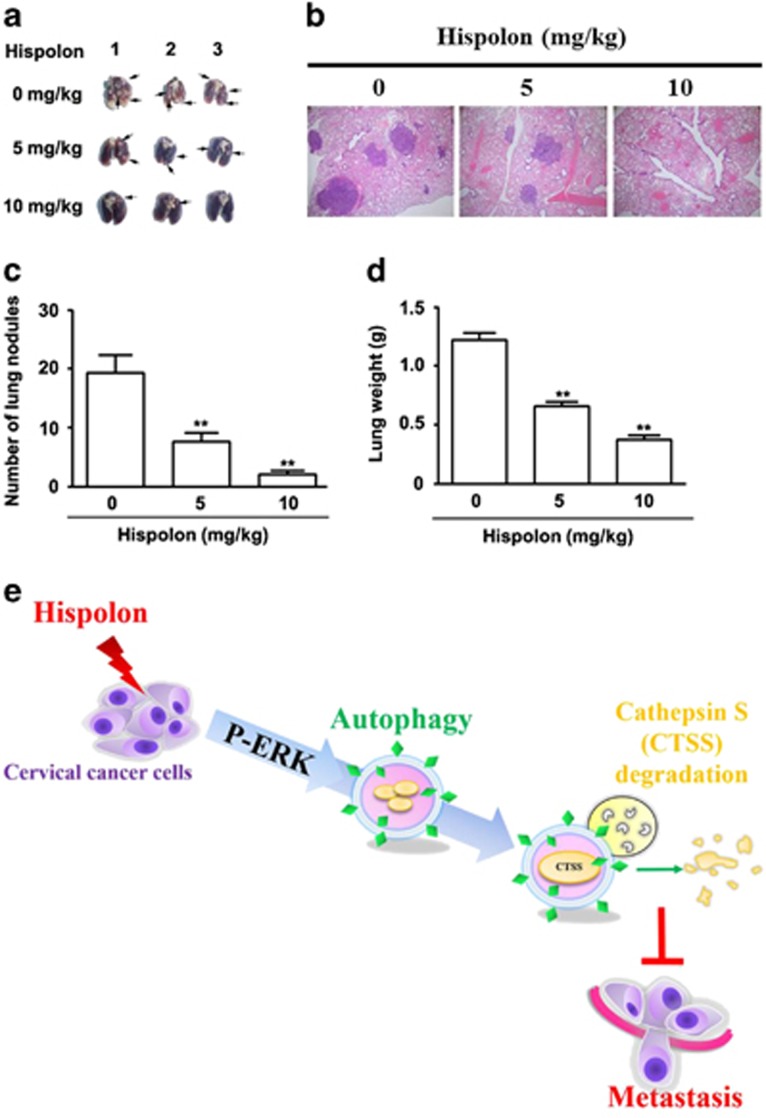
Hispolon suppressed cell metastasis *in vivo*. (**a**) For the xenograft, a total of 2 × 10^6^ cells were injected subcutaneously into the left flank. Photographs of lungs of the mice in each group (*n* = 5/group) for 4 weeks. (**b**) Photographs of lungs of the mice from each group were sectioned for evaluation of lung metastasis after H&E staining. (**c**) There were lung metastatic colonies in the hispolon-treated group significantly fewer than in the control group. (**d**) Average lung weight of the mice from each group were removed and calculated. **P<0.01. (**e**) The schematic representation of anti-metastasis effects of hispolon in human cervical cancer cell

To examine the metastasis-inhibiting ability of hispolon in SiHa cells, three groups of mice (five per group) were intravenously injected with SiHa cells in the tail vein. After the indicated number of days, the mice were killed, and the metastatic nodules on the lung surfaces were counted. As shown in Figure 7a, imaging showed that the incidence of lung metastasis was low in mice after hispolon treatment. Hematoxylin and eosin staining suggested that the nodules on the surface of the lungs were metastatic tumors (Figure 7b). Moreover, after hispolon treatment, SiHa cells established statistically fewer lung metastatic colonies than control cells (*P*<0.05, Figure 7c). Additionally, the lung weight was lower in the hispolon-treated group than in the control group (Figure 7d). These results indicate that hispolon inhibited cervical cancer tumor metastasis *in vivo*.

## Discussion

In recent years, natural products isolated from plants have been reported to exhibit anticancer effects.^25, 26, 27, 28^ The present study demonstrated that hispolon, a pure compound, may significantly inhibit the migration and invasion of HeLa and SiHa cells, suggesting a potential role of this compound in the treatment of metastatic cervical cancer. Degradation of the basement membrane by cathepsins plays a crucial role in the development of cancer metastasis. The Cathepsin family has critical roles in extracellular matrix degradation in cancers.^29, 30, 31, 32^ In the present study, treatment with hispolon and Z-FL-COCHO suppressed CTSS expression and the cell migration ability of the cervical cancer cell line. This finding suggests that hispolon inhibits metastasis by reducing the activity of CTSS.

Hispolon induced appropriate autophagy pathways and significantly increased LC3-II accumulation in our study. Cleaved GFP, a marker of autophagic flux in GFP–LC3 transfected cells,^9^ played a critical role in identifying two different methods to monitor autophagy. Free GFP released from GFP-LC3 and endogenous LC3-II turnover blocked by chloroquine indicate that hispolon induced the normal flux of autophagy (complete autophagy) in our model (Figures 3f and g). Normal flux suggests that the cargos in autophagosomes, including LC3-II, are lysed and recycled.^11^ LC3 and Beclin-1 are the two essential proteins of autophagy. Through the siRNA system, we further proved the direct relationship between autophagy and the anti-metastatic effects of hispolon (Figures 4a and b). Notably, CTSS interacted and colocalized with LC3, the autophagosome protein. These data demonstrated that hispolon induced CTSS ubiquitination and following degradation through autophagy/lysosome pathway. We suggested that ubiquitination of CTSS may interfere in the delivery mechanism of CTSS and lead to autophagic degradation after hispolon treatment. Therefore, CTSS procced to ubiquitination to degradation via autophagy.

Autophagy plays an essential role in the type of cell death accompanied by the large-scale autophagic vacuolization of the cytoplasm.^33^ Recently, an increasing number of studies have reported on the relationship between autophagy and metastasis. Autophagy inhibition may suppress metastasis as a result of the survival mechanisms of autophagy in cancer cells.^34, 35^ Here, our data provide an alternate perspective that activated autophagy may inhibit metastasis because of the degradation of the proteins promoting cell migration. Tuloup-Minguez *et al.* also suggested that low levels of autophagy do not cause cell death but reduce cell migration.^36^

The MAPK phosphorylation pathway is involved in many cellular processes such as cell growth, differentiation, proliferation, apoptosis, and migration.^22^ Our results demonstrate that hispolon activated ERK phosphorylation in cervical cancer cells. The MEK inhibitor U0126 was used to show that hispolon induced autophagy to inhibit metastasis through the p-ERK pathway. Consistently, Zhi *et al.* reported that quercitrin, a plant-derived flavonoid compound, can promote autophagy through ERK activation.^37^ Moreover, Yeh *et al.* reported that honokiol can induce the autophagy of neuroblastoma cells through activation of the ERS/ROS/ERK1/2 signaling pathways and the suppression of cell migration.^38^ Cagnol *et al.* and Wang *et al.* suggested that the ERK pathway is also a noncanonical method of regulating autophagy,^39, 40^ demonstrating that anti-metastatic agents have favorable and unfavorable consequences.^41^ These results suggest that the ERK/CTSS pathway is involved in the hispolon-mediated inhibition of migration and invasion of cervical cancer cells.

In this study, we found that hispolon reduces Cathepsin S expression via autophagy/lysosome degradation pathway. Cathepsins are lysosomal proteases with different half-lives. Previous study has shown that the half-lives of pro-Cathepsin S in normal cells and tumor cell lines were 1 and 2 h, respectively. Half-life of mature-Cathepsin S was 16–18 h, suggesting that mature-Cathepsin S is more stable than pro-Cathepsin S. Nissler *et al.* found that E-64, a cysteine protease inhibitor, can inhibit cleavage and degradation of Cathepsin L. These publications provide the evidences that Cathepsins degradation in lysosome occurs naturally.^42, 43^ In our results, hispolon inhibited both pro- and mature-Cathepsin S in cervical cancer cells by autophagy (Figure 5b and Supplementary Figure S2). Taken together, we suggested that hispolon-induced ubiquitination leads Cathepsin S to autophagosomal sequestration and lysosomal degradation.

In conclusion, this is the first scientific report describing that hispolon inhibits cervical cancer invasiveness through autophagy by reducing the production of tumor metastasis-related proteins. Our data suggest that hispolon suppressed the metastatic ability of cervical cancer cells through autophagy via the ERK pathway (Figure 7e). These results indicate that hispolon may be an influential pharmaceutical compound with anti-metastatic effects in cervical cancer. This study also demonstrated the crucial role of autophagy. Accordingly, we hope that the findings of this study merit further investigation of the applicability of hispolon for clinical treatment.

## Materials and methods

### Cell lines and culture

HeLa and SiHa, human cervical cancer cell lines were cultured in Dulbecco’s modified Eagle’s medium (DMEM) (Gibco, Gran Island, NY, USA) supplemented with 10% fetal bovine serum (FBS) (Hyclone Laboratories, Logan, UT, USA) and 100 ng/ml each of penicillin and streptomycin (Sigma, MO, USA). All cell lines were cultured at 37 °C in a humidified atmosphere of 5% CO_2_.

### Cell viability assay and treatment

Hispolon, ⩾98% (HPLC), the yellow solid powder was purchased from Enzo Life Sciences, NY, USA. Stock solution of hispolon was made at 25, 50, 100 mM concentration in DMSO. HeLa and SiHa cell lines were treated with hispolon (25, 50, 100 mM) in the final concentration and all treatments of DMSO was consistently less than 0.1%. The stock solution was protected from light and stored at −20 °C. We seeded 7 × 10^4^ of each HeLa cells onto 24-well plates and pretreated them with hispolon (0, 25, 50, 100 *μ*M) for 24 h. After that we removed the medium carefully and washed 1 × PBS then cells continuously treated with 0.5 mg/ml MTT (Sigma) at 37 °C in 5% CO_2_ for 4 h. The viable cells were detected by spectrophotometrically at 563 nm (Beckman Spectrophotometer DU640).

### Cell migration and invasion assay

After the cells were treated with different concentrations of hispolon, we collected the cells by Trypsin (Gibco, Gran Island, NY, USA) and the tumor metastasis assay *in vitro* was tested by Boyden chamber (NeuroProbe, Inc., MD, USA). Treated cells in 0.5% FBS medium were loaded into the well of the chamber at the upper part and incubated for 24 h (migration), 48 h (invasion) at 37 °C. Compared to cell migration, the invasion membrane filters were coated with 10 *μ*l Matrigel (25 mg/50 ml; BD Biosciences, Bedford, MA, USA) and air-dried for 5 h in a laminar flow hood.^28, 44^ The migration cells were fixed by methanol and stained with Giemsa and counted by light microscopy.

### Western blot assay

7 × 10^5^ cells were seeded onto 6 cm dish and treated with hispolon, then total cell lysates were collected with 200 *μ*l of lysis buffer (50 mM Tris-HCl, pH 7.5, 0.5 M NaCl, 5 mM MgCl_2_, 0.5% Nonidet P-40, 1 mM phenylmethylsulfonyl fluoride, 1 *μ*g/ml pepstatin, and 50 *μ*g/ml leupeptin) on ice. After centrifuged at 13 000 × *g* at 4 °C for 30 min, the protein lysates were separated by 12% agarose gel and transferred onto a nitrocellulose membrane then blocking with 5% non-fat milk in Tris-buffered saline (20 mM Tris, 137 mM NaCl, pH 7.6) for 1 h in room temperature and overnight with first-antibodies in 4 °C and second-antibodies for 1 h in room temperature. Anti-pERK (Cell Signaling Technology, MA, USA), anti-ERK (Cell Signaling Technology), anti-pJNK (Cell Signaling Technology), anti-JNK (Cell Signaling Technology), anti-pFAK 397 BD Biosciences), anti-pFAK 925 (Cell Signaling Technology), anti-FAK (BD Biosciences), anti-pAkt (Cell Signaling Technology), anti-Akt (BD Biosciences), anti-pp38 (Cell Signaling Technology), anti-p38 (BD Biosciences), anti-pro-Cathepsin S (GeneTex), anti-mature-Cathepsin S (Abcam, Cambridge, MA, USA), anti-*β* actin (Novus Biologicals, ON, Canada), anti-uPA (Santa Cruz, Santa Cruz, CA, USA), anti-MMP-9 (Millipore, Bedford, MA, USA), anti-LC3B (Cell Signaling Technology), anti-p53 (Dako Corp., Carpinteria, CA, USA), anti-Beclin 1 (Cell Signaling Technology), anti-GFP (Santa Cruz), anti-p62 (GeneTex), anti-Ubiquitin (Millipore).

### Co-immunoprecipitation assay

1.8 × 10^6^ cells were seeded onto 10 cm dish and treated with different concentrations of hispolon, then the total cell lysates were collected with 500 *μ*l of NETN buffer (NaCl 150 mM, EDTA 1 mM/pH 8, Tris 20 mM/pH 8, NP-40 0.5% added with 1 mM PMSF, 1 mM NaF, 1 mM Na_3_VO_4_, and 2 *μ*g/ml Aprotinin). After sonication they were centrifuged at top speed at 4 °C for 30 min. Collected supernatant and quantify protein amount then added 20 *μ*l Protein A per sample for pre-clearing rotated at 4 °C for 1–3 h. Centrifuged at 3000 r.p.m. at 4 °C for 2 min and discard supernatant then added wash buffer NETN up to 1 ml/eppendorf and inverted up and down for few times. As the same step that centrifuged at 3000 r.p.m. at 4 °C for 2 min and discard supernatant and repeat three times. Resuspended in 25 *μ*l (2 × SDS sample buffer and 1/20 volume 2-ME, half protein a volume) and boiled at 95 °C for 5 min then spin down at top speed for 15–30 s. Collected supernatant carefully and loaded into SDS-PAGE. Then the same way like transfer in western blot as we have described.

### Detection of AVO development

After treatment, the cells were washed by 1 × PBS three times, staining by 1 *μ*g/ml acridine orange (Sigma, MO, USA) and dilution in PBS containing 5% FBS for 10 min and were observed under a red filter fluorescence microscope.

### GFP-LC3 transfection and GFP-LC3 dot formation

The cells were seeded onto 24-well plates and cultured overnight then transfected with 1 *μ*g working GFP-LC3 per well. After incubated for 24 h, the cells were treated continuously with hispolon (100 *μ*M) for 24 h. The GFP-LC3 dots in cells analyzed by fluorescence microscope.

### Small interfering RNA (siRNA) system

The cells were seeded onto 6 cm plates. After cultured overnight, we transfected the siRNA into cells and working for 48 h at 37 °C. Removed the reagent carefully and washed with 1 × PBS buffer then treated the cells continuously with hispolon (100 *μ*M) for 24 h. The effects of siRNA detected by western blot. Cathepsin S-siRNA (Ambion, Austin, TX, USA), LC3-siRNA (Cell Signaling Technology), Beclin 1-siRNA (Cell Signaling Technology), ERK-siRNA (Sigma).

### Inhibitor system

The cells after seeded and incubated then pre-treated with inhibitor for 1 h and continuously co-treated with hispolon for 24 h. The results analyzed by western blot. U0126 (Promega, Madison, WI, USA), Z-FL-COCHO (Millipore), Chloroquine diphosphate salt (Sigma), 3-Methyladenine (Sigma), Bafilomycin A1 (Sigma).

### Immunofluorescence staining

The cells were seeded onto coverslips in 24-well plates. After incubated overnight, the cells were treated with hispolon for 24 h and then washed with 1 × cold PBS (pH 7.4) twice. Incubate the samples with 4% paraformaldehyde in 1 × PBS at room temperature for 15~20 min, followed by 1 × PBS washing three times. Then incubate the sample with cold 0.5% TritonX-100 in 1 × PBS for 10 min, and blocking with 1% BSA in PBS for 1 h. Incubate the samples with first antibody solution (suitable diluted rate in 1% BSA/1 × PBS, 50~80 *μ*l/slide) for overnight under the light protection. Then incubate the samples with suitable secondary antibody prepared in 1% BSA/1 × PBS at room temperature for 1 h and stain the cell nuclear with DAPI (1000 × in PBS) for 5~10 min. Wash the samples with 1 × PBS 3 min/per wash. The results were analyzed by a confocal microscope.

### Wound healing assay

HeLa and SiHa cells were seeded onto 6-well plates and incubated overnight. Then the cells were scratched a line by tips then treated with different concentrations of hispolon. We observed the ability of cell healing on different times by microscopy.

### *In vivo* tumor xenograft model

SiHa cells (2 × 10^6^) in 0.1 ml of DMEM were subcutaneously injected into the right flank of NSG mice. After transplantation, the tumor size was measured using calipers and the tumor volume was estimated by the following formula: tumor volume (mm^3^)=length × width^2^ × 1/2. Once the tumor reached a volume of 250 mm^3^, animals began receiving intraperitoneal (i.p.) injections of DMSO or hispolon (5 and 10 mg/kg) in DMSO five times per week for 2 weeks. Each mouse was weighed every 2 days to evaluate the side effects of administration, and the tumor size was measured. Mice were killed at 15 days after the hispolon or DMSO injections. Tumor masses were then excised for immunohistochemical staining.

### Cathepsin S activity assay kit (Fluorometric)

The Cathepsin S activity was analyzed using a Cathepsin S activity assay kit (cat. no. ab65307, Abcam, Cambridge, UK). After treatment with hispolon in various time course, the prepared samples were added to an ELISA plate, according to the manufacturer’s instructions. The Cathepsin S activity was quantified after reading the absorbance of each well at 400 nm in a microtest plate spectrophotometer (STNERGY/H4, BioTek Instruments, Inc., Winoosi, VT, USA).

### Statistical analysis

Statistically significant differences were calculated using the Student’s *t*-test (SigmaPlot 10.0, Jandel Scientific, and San Rafael, CA, USA). Significance was set at *P*<0.05. The values are the means±standard deviation (S.D.) of at least three independent experiments.

## Publisher’s Note

Springer Nature remains neutral with regard to jurisdictional claims in published maps and institutional affiliations.
