# Supplementary material for: Hispolon suppresses metastasis via autophagic degradation of cathepsin S in cervical cancer cells
Source: Cell Death Dis. 2017 Oct 5;8(10):e3089–. doi: 10.1038/cddis.2017.459 (PMC5680581; doi:10.1038/cddis.2017.459)
Supplement: Supplementary Information [file cddis2017459x1.docx]

**Hispolon Suppresses Metastasis via Autophagic Degradation of Cathepsin S in Cervical Cancer Cells**

Min-Chieh Hsin^1^, Yi-Hsien Hsieh^2^, Po-Hui Wang^1,3^, Jiunn-Liang Ko^1^, I-Lun Hsin^4^, Shun-Fa Yang^1,5,^*

^1^Institute of Medicine, Chung Shan Medical University, Taichung, Taiwan

^2^Institute of Biochemistry, Microbiology and Immunology, Chung Shan Medical University, Taichung, Taiwan

^3^Department of Obstetrics and Gynecology, Chung Shan Medical University Hospital, Taichung, Taiwan

^4^Inflammation Research & Drug Development Center, Changhua Christian Hospital, Changhua, Taiwan

^5^Department of Medical Research, Chung Shan Medical University Hospital, Taichung, Taiwan

***Address correspondence to:** Shun-Fa Yang, PhD., Institute of Medicine, Chung Shan Medical University, 110 Chien-Kuo N. Road, Section 1, Taichung 402, Taiwan. Telephone: +886-4-24739595 ext. 34253; Fax: +886-4-24723229; E-mail: ysf@csmu.edu.tw

**Running Title**: Hispolon inhibits cervical cancer metastasis

**Competing Interests**: The authors have declared that no competing interests exist.

**Supplementary Figure**


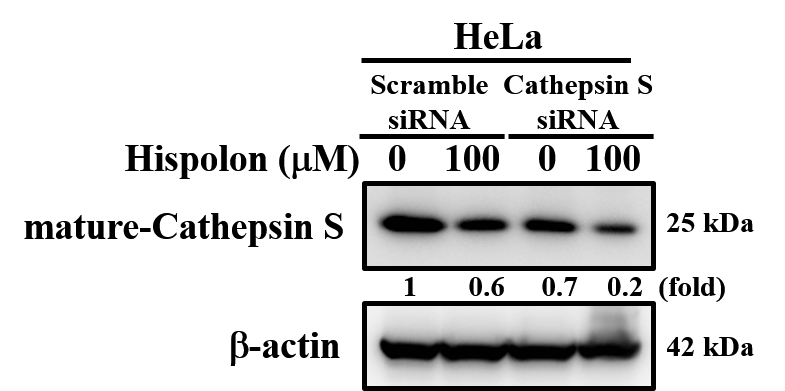


**Figure S1.** Hispolon and Cathepsin S silencing suppressed mature-Cathepsin S protein level. HeLa cells were transfected with CTSS siRNA for 48 h and treated with hispolon then analyzed by Western blot assay.

**
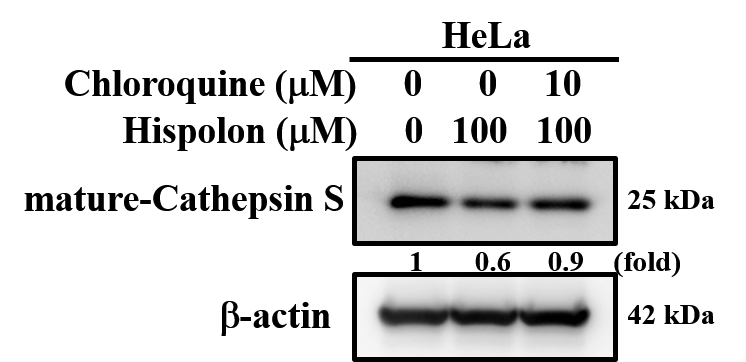
**

**Figure S2.** Hispolon suppressed mature-Cathepsin S via autophagy. HeLa cells were pre-treated with chloroquine for 1 h and co-treated with hispolon for 23 h then analyzed by Western blot assay.
