# Supplementary figures and images for: Hispolon suppresses metastasis via autophagic degradation of cathepsin S in cervical cancer cells
Source: Cell Death Dis. 2017 Oct 5;8(10):e3089–. doi: 10.1038/cddis.2017.459 (PMC5680581; doi:10.1038/cddis.2017.459)

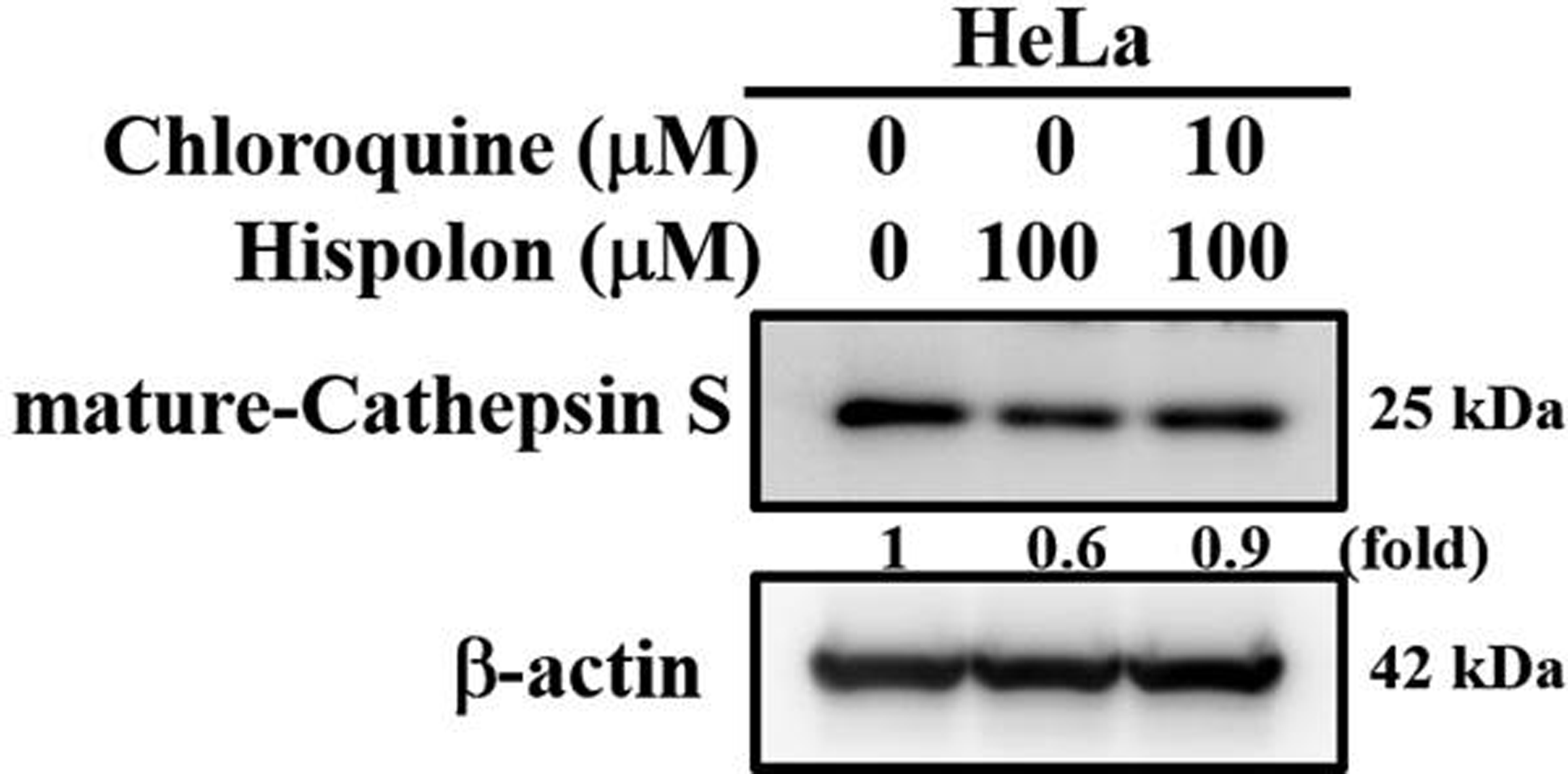

Supplement: Supplementary Figure 1 [file cddis2017459x2.tif]
